# Ectodermal Dysplasia: A Case Report

**DOI:** 10.7759/cureus.21184

**Published:** 2022-01-12

**Authors:** Hussein A Alshegifi, Abdulmajeed M Alamoudi, Abdullah Alrougi, Hassan Alshaikh, Awadh Alamri, Aiman M Shawli

**Affiliations:** 1 Medicine and Surgery, King Saud Bin Abdulaziz University for Health Sciences College of Medicine, Jeddah, SAU; 2 Internal Medicine, King Saud Bin Abdulaziz University for Health Sciences College of Medicine, Jeddah, SAU; 3 Medicine, King Saud Bin Abdulaziz University for Health Sciences College of Medicine, Jeddah, SAU; 4 Dermatology, King Abdulaziz Medical City, Western Region, Jeddah, SAU; 5 General Pediatrics and Pediatric Genetics, King Abdulaziz Medical City, Western Region, Jeddah, SAU

**Keywords:** developmental malformation, ectodermal layers, exocrine glands, rare genetic disorder, x-linked genetic diseases, ectodermal dysplasia

## Abstract

Ectodermal dysplasia (ED) is a hereditary genetic disorder that manifests a variety of deformities in one or more of the ectodermal derivatives. Ectodermal derivatives originate from ectodermal layers during embryonic development, such as skin, nails, hair, teeth, and exocrine glands. Over 150 variants of ED are reported in the literature. It has an incidence of seven in every 100,000 live births. There are two types of ED, which are hypohidrotic (anhidrotic) and hydrotic. The types are classified according to the degree of function of the sweat glands. This report discusses the case of a 13-month-old Saudi girl with typical features of ectodermal dysplasia who presented to a dermatology clinic.

## Introduction

Ectodermal dysplasia (ED) is a rare hereditary disorder that forms a diverse group of inherited diseases that demonstrate primary developmental defects. It is characterized by congenital defects in two or more ectodermal structures, including skin, nails, teeth, hair, or sweat glands. It can also affect the development of other embryonic ectodermal organs such as parts of the eyes, ears, neural and adrenal tissues to various degrees [1]. Fortunately, ectodermal dysplasia is considered to be a relatively rare disorder, with an estimated incidence of around seven cases per 100,000 people [2]. There are two main types of ectodermal dysplasia based on the degree of sweat gland function. The first type is an X-linked hypohidrotic form that is characterized by a classical triad of hypodontia, hypohidrosis, and hypotrichosis. In contrast to that, the second type is the hydrotic type that is characterized by a lack of sweat gland involvement and is inherited as an autosomal trait [1]. The pathogenesis of ectodermal dysplasia and the molecular basis of many of these disorders are still unknown [3]. Recent reports have shown that loss-of-function variants within the thrombospondin-type laminin G domain and epilepsy-associated repeats (TSPEAR) gene have recently been related to ectodermal dysplasia [4]. TSPEAR mutations or down-regulation resulted in altered expression of genes known to be regulated by the NOTCH signaling pathway and to be involved in murine hair and tooth development. Further functional evidence is needed to evaluate this phenotypic association as this gene plays a critical, previously unrecognized role in human tooth and hair follicle morphogenesis [3]. We aim to report this rare case using theoretical concepts from our discipline and recommend a course of action for similar cases in the future.

## Case presentation

**Figure 1:**
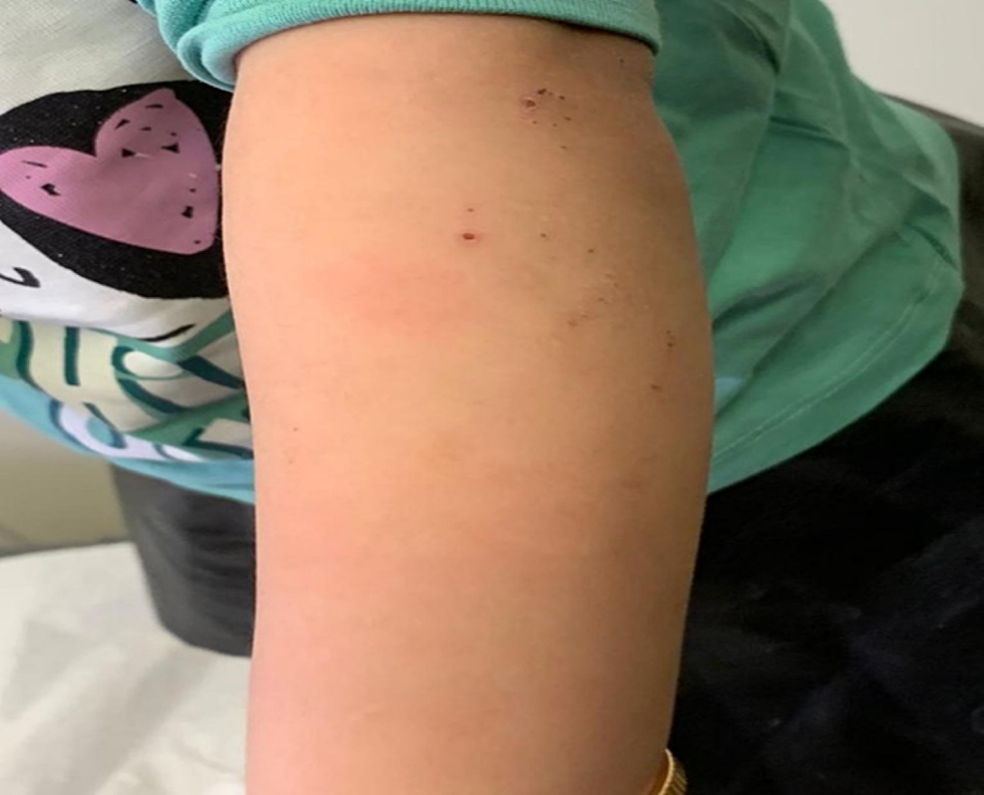
Photograph of the left forearm The skin view showing excoriated papules, scratch marks, and generalized xerosis all over the left forearm.

**Figure 2:**
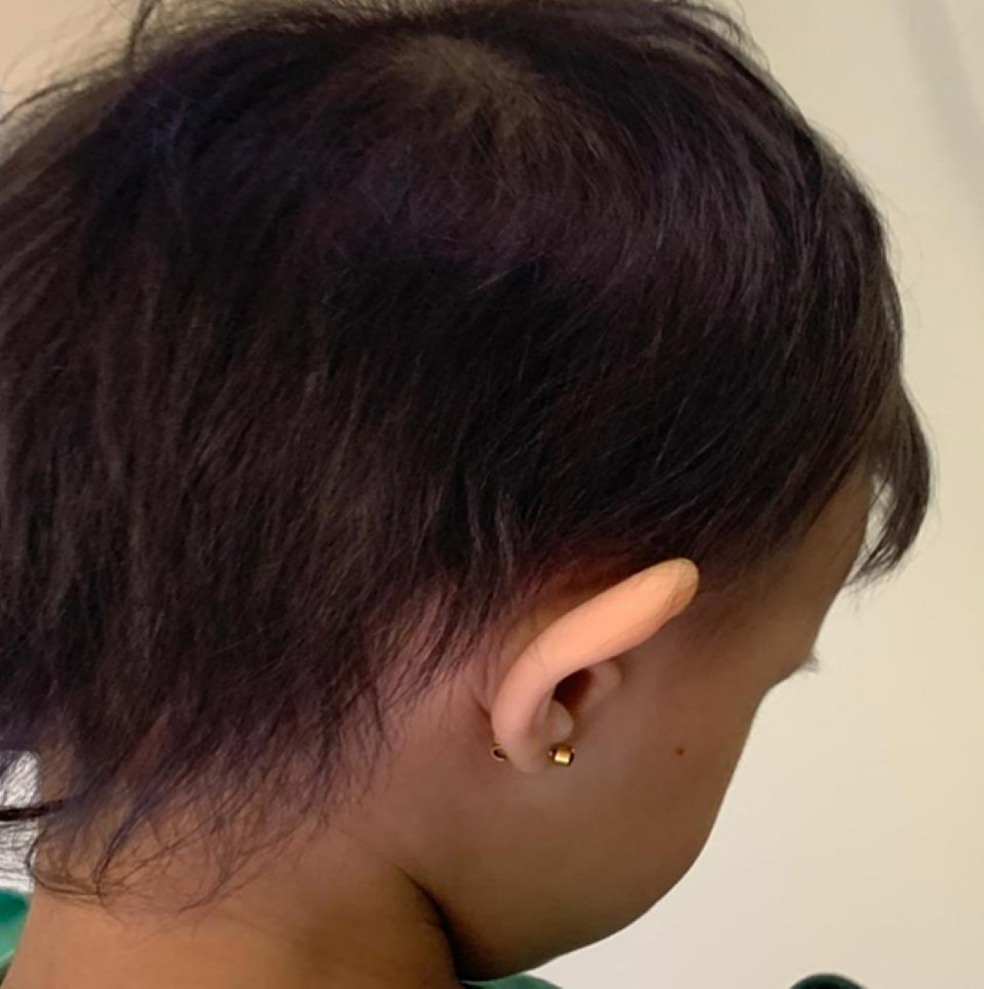
Hair photograph Sparse, fair, lightly pigmented and wiry hair with preserved eyelashes and eyebrows (not visualized).

A 13-month-old Saudi girl, as a result of a non-consanguinity marriage, was born at term to a healthy pregnant mother, apart from hypothyroidism and gestational diabetes mellitus, which required levothyroxine 75 mcg and dietary modifications. She is the second child with a positive family history of ectodermal dysplasia in her 11-year-old sister. She is growing normally with no history of developmental delay or family history of cleft palate or lips, abnormal nails, or other abnormalities. The baby girl was presented by her mother to the dermatology clinic at King Abdulaziz Medical City Hospital in Jeddah, Saudi Arabia with a chief complaint of skin dryness and itching all over her body. Upon clinical examination, the skin showed excoriated papules, scratch marks, and generalized xerosis with hypohidrosis all over the legs and forearms (see Figure 1). Moreover, hair examination revealed hypotrichosis that was noticed in the scalp with preserved eyelashes and eyebrows (see Figure 2).

**Figure 3:**
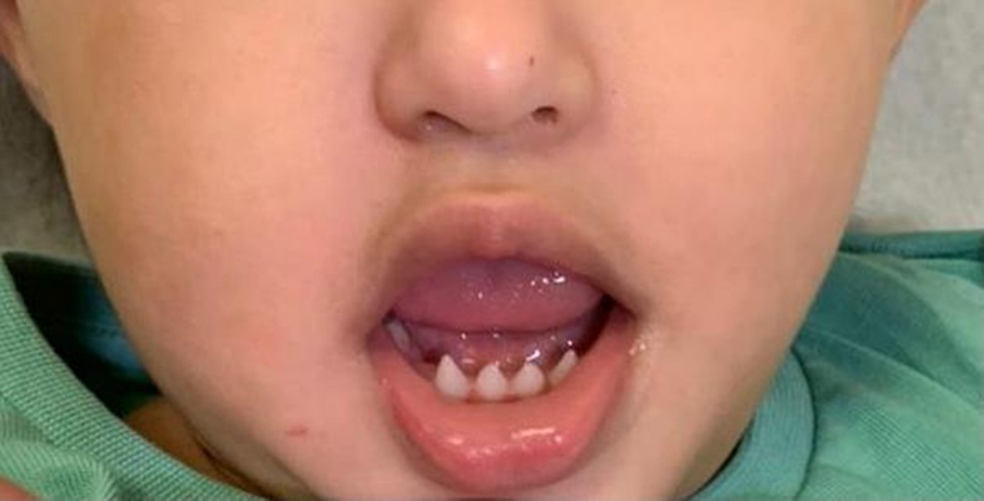
Photograph of the mouth Everted lips with conical-shaped incisors, without first molar teeth in the lower arch.

The fingers were normal-shaped, along with nail appearance. On ear examination, the ear was full of wax with difficult visualization. The patient had a typical face characterized by a flat nasal bridge and everted lips. Furthermore, intraoral examination showed a dry mucous membrane. In the upper arch, the central incisor teeth appeared abnormally conical in shape. It also showed only the second molar teeth, with no first molar teeth. Regarding the lower arch, it showed conical-shaped incisors, without first molar teeth too (see Figure 3).

Molecular genetic analysis of whole exome sequencing (WES) was performed for this patient and identified two different heterozygous variants as the following:

1. Heterozygous variant c.1726_1728delinsTT p.(Val576leufs*38) in TSPEAR (OMIM:612920) which leads to a frameshift, resulting in a premature stop code and subsequent mRNA degeneration (nonsense-mediated decay) or truncation of the protein. This variant has already been described in the literature in patients with tooth agenesis (PMID: 27736875, 30046887, 22678063). Considering the available information the variant is classified as pathogenic.

2. Another heterozygous variant c.1325A>C p.(Asn442Thr) in TSPEAR (OMIM:612920) which leads to an amino acid exchange. Nineteen out of 22 bioinformatic in silico programs predict a pathogenic effect for this variant. To the best of our knowledge this variant has not been described in the literature so far (HGMD 2020.4). Considering the available information the variant is classified as likely pathogenic. Parallel analysis of parental WES data confirmed compound heterozygosity of the detected variants.

## Discussion

ED is a term for rare heterogeneous hereditary disorders that are characterized by abnormal development of two or more embryonic ectodermal tissues at a time. ED is a congenital, non-progressive disease that can be isolated where the defect is in the ectodermal structures alone or it can be a syndrome in which the defect is in ectodermal origin in association with other anomalies. X-linked recessive hypohidrotic ectodermal dysplasia and hidrotic ectodermal dysplasia are the most common types of ectodermal dysplasia [5]. There is a wide variety of hair manifestations, ranging from sparse, short, fine, dry, to complete absence of hair and decreased density of eyebrows [6]. In this present report, our patient has some similarities of hair manifestations of ectodermal dysplasia that include little or no hair distribution on the scalp with preserving eyelashes and eyebrows, in comparison to other case reports that showed a scanty hair on the scalp, eyebrows, and lashes too [1]. Face manifestations include a saddle nose, low-set ears, and thick everted lips. Some dental manifestations are hypodontia, complete anodontia, delayed eruption of permanent teeth, conical or pegged teeth [6]. Similar to other case reports, our case exhibits the same face and dental manifestations which include a flat nasal bridge and everted lips. In addition to that, it also exhibits a dry mucus membrane, partial hypodontia with conical shape central incisors teeth [5]. In comparison to other cases, excoriated papules, scratch marks, generalized xerosis with hypohidrosis all over the legs and forearms are seen in our patient [1]. Due to various consequences such as failure to thrive, lung infections, and hyperthermia, mortality in children with hypohidrotic ED can reach 30% in the first three years of life. As a result, the treating physician should pay extra attention to newborns and young children. Life expectancy is typical after three years of life [2]. ​Management of ED depends on the organ affected and early intervention is of paramount importance to an effective and successful management of such a disease [5]. Heat exposure must be closely managed with physical cooling methods such as frequent drinking of cold drinks, wearing special cooling vests to prevent heat generation during activities. Early dental treatment can enhance the function and appearance of the teeth. Under the supervision of an orthodontist, bone grafting or sinus lift treatments, dental implants, and dental prostheses are strongly recommended. 3% minoxidil has recently been attempted to promote hair growth in ectodermal dysplasia patients. Emollients are the preferred therapy for xerosis as well as wound care with topical and systemic medications [7]. In this case, the skin was managed conservatively with hydrocortisone, antihistamine, and moisturizing cream. Regarding the oral manifestations, fluoride was applied and the patient was educated about preventive dental care and bottle-feeding discontinuation and their effects on teeth. Moreover, this disease needs close monitoring and frequent follow-up as a part of treatment plan.

## Conclusions

ED is a term for a group of diseases that affect the skin, exocrine glands, teeth, and hair. It is an inherited genetic disorder that may impact many organs and cause variable manifestations. Because of the undesirable appearance and malfunction of orofacial structures, patients with ED have poor psychological and physiological development. In order to detect this illness as soon as feasible, primary provider education is critical. As many body organs are impacted, interpersonal communication between the pediatrician, dermatologist, dentist, and other providers may be essential. To prevent problems in such individuals, parents must be adequately informed on the condition and day-to-day routine treatment.
